# Quality of life and caregiving burden associated with parenting a person with Duchenne/Becker muscular dystrophy in Poland

**DOI:** 10.1186/s13023-024-03481-7

**Published:** 2024-11-30

**Authors:** Jan Domaradzki, Dariusz Walkowiak

**Affiliations:** 1 Department of Social Sciences and Humanities, Poznan University of Medical Sciences, Rokietnicka 7, Poznań, 60-806 Poland; 2 Department of Organization and Management in Health Care, Poznan University of Medical Sciences, Poznań, Poland

**Keywords:** Becker muscular dystrophy, Caregiving burden, Duchenne muscular dystrophy, Family caregivers, Quality of life

## Abstract

**Background:**

Duchenne and Becker muscular dystrophies (DBMD) are rare progressive, X-linked diseases of muscle wasting characterised by the early onset and prognosis of premature death. The aim of this study was to evaluate the impact of providing care for a person with DBMD on caregivers’ quality of life, perceived burden and financial well-being.

**Results:**

202 Polish caregivers of a person with DBMD were included and completed a self-administered, computer-assisted online survey. Results show that DBMD affects the quality of life of caregivers who score significantly lower than the national average in every domain: 85.1% of DBMD caregivers scored below the national average in the physical health domain; in the environmental domain 83.7%; in the social relationships domain 82.2%; and in the psychological domain 72.3%. It also demonstrates that DBMD is a source of severe burden (mean score of 35.3). Finally, we report that while some dimensions of respondents’ quality of life and perceived burden were associated with patients’ age and caregivers’ educational status, financial well-being was the most important predictor of respondents’ quality of life and caregiving burden.

**Conclusions:**

To improve DBMD caregivers’ quality of life and alleviate their burden, future intervention programs should promote resiliency and active coping and develop a social support system and respite care. Additionally, it is crucial to provide caregivers with adequate financial resources to fulfil their needs.

## Background

Caring for a person with a rare disease (RD) impacts the entire family, including caregivers’ physical and mental health, family dynamics, social relations and financial burden [1–8]. However, although caring for a person with a chronic disease requires adopting the psychosocial model of care that includes the entire family [9, 10], a recently adopted in Poland new *Plan for Rare Diseases for 2024–2025* concentrates on the clinical dimension of these diseases and addresses mainly patients’ needs, including the improvement of access to specialized centres, new diagnostics tests and treatment options for RDs (i.e. medicines, medical devices and special nutritional products) [11]. While all these solutions are of crucial importance, the problem is that the *Plan* emphasizes patients’ clinical needs but inadvertently ignores the psychological, social, economic and educational needs of family caregivers, which go beyond the purview of state health and social policy. Meanwhile, it was demonstrated that providing care for RD patients results in a decline in the caregivers’ physical and mental well-being, emotional and financial burdens and a reduction in caregivers’ quality of life [1–8]. Consequently, as a person with RD profoundly impacts caregivers, modern healthcare policies stress the need to adopt family-based and family-centred approaches and strategies for RDs [12, 13]. However, although all family members should receive complete and integrated support, the *Plan* fails to provide them with the necessary resources, support and care [14].

This is particularly important for conditions like Duchenne muscular dystrophy (DMD) and Becker muscular dystrophy (BMD) since psycho-social costs and caring obligations in DBMD rise over time [15–21]. Especially since, due to improved care and constant progress in treatment, the number of adult persons with these conditions is growing, making the idea of DBMD as a mere “paediatric” disease being revised. Thus, transitioning from childhood to adulthood is challenging [22–24], so patients and their families require constant psychosocial management [25, 26].

Duchenne muscular dystrophy is a rare progressive, X-linked disease of muscle wasting caused by mutations that change how the dystrophin protein is produced, which, in turn, damages the healthy growth of muscle tissue [27, 28]. The prevalence of DMD is estimated at less than 10 cases per 100,000 males [29–31] and is characterised by the early onset and prognosis of premature death. While DMD is usually diagnosed at the age of 3–4, its early signs include delayed sitting, trouble getting up and standing independently, and difficulties in speaking, which are followed by the malfunction of the respiratory and cardiac systems. Additionally, some individuals experience behavioural and cognitive impairment, including intellectual and learning disabilities, autism spectrum disorders and attention hyperactivity disorder [32–34]. The rapid progress of muscle weakness results in wheelchair use by age 10–12 and death approximately at the age of 20 to 30, usually due to respiratory issues or heart failure [35, 36]. Becker muscular dystrophy is a less common variant of the two diseases, with an estimated prevalence of one in 16–30,000 males [30]. While mutations in the same gene cause BMD [29, 30], it is more heterogeneous and a milder variant, with a later onset, slower progression, less severe muscle weakness, and higher life expectancy of approximately 40 to 50 years [37].

Previous research on the needs and challenges of caring for a person with DBMD described important components of caregivers’ elevated stress and decreased quality of life [18, 20, 38–40], which include impaired caregivers’ health, pain, poor sleep patterns, the risk of anxiety and depression related to unfavourable diagnosis and anticipated feelings of loss [18, 41–44]. What is equally important is that DBMD caregivers often report higher levels of depression, anxiety and emotional strain than the general population. Moreover, while their quality of life is lower compared with the general population and caregivers of healthy children, they experience higher levels of caregiving burden and financial strain [15, 17, 18, 39]. Similar to other diseases with an X-linked pattern of inheritance [45, 46], due to their carrier status, DBMD mothers often experience feelings of guilt and self-blame [40, 47, 48]. DBMD parents are also at higher risk for sexual dysfunction [49], and experience disrupted family and social life. Finally, DBMD caregivers struggle with high direct and indirect medical costs and indirect costs resulting from caregivers’ impaired ability to work, loss of productivity, absenteeism and a general financial burden that is up to 10 times higher per patient compared with individuals without the disease [16, 21, 50–52]. At the same time, it is teenage DBMD caregivers who report a more significant influence and higher burden [53].

Although the caregiver burden associated with neuromuscular illnesses has been the subject of numerous research in recent decades, still little is known about the experiences of Polish DBMD caregivers. Meanwhile, one of the few Polish studies conducted demonstrated that caring for a child with DMD negatively affects mothers’ life satisfaction and meaning of life, which, in turn, influences their self-actualization and mental well-being, quality of life and social functioning [54, 55]. Therefore, this study aimed to assess the quality of life, perceived burden and financial well-being associated with caregiving for a person with DBMD. Specifically, we sought to answer the following questions: (1) How do DBMD affect caregivers’ quality of life? (2) How do DBMD affect perceived strain in caregivers? (3) Do DBMD affect caregivers’ financial well-being? (4) What is the association between caregiver burden, quality of life and financial well-being?

## Methods

### Study design

As a part of a broader project on the quality of life and caregiving burden of parents with RDs, this study analyses the experiences of informal caregivers of persons with DBMD. Due to a lack of a Polish patient registry, we contacted several advocacy organizations and support groups for DBMD patients and their families, which helped recruit caregivers. The study was designed as a self-administered, anonymized, computer-assisted online survey to assess caregivers’ quality of life, financial well-being and caregiving burden. The design of the study followed the Declaration of Helsinki (revised in 2000) [56] and was approved by the Poznan University of Medical Sciences Bioethics Committee (KB – 228/24).

To ensure the reliability of the information provided in an electronic survey, the survey questions were written in plain, clear and unambiguous language, and each item was provided with appropriate and inclusive response options to cover as many responses as possible. Additionally, the questionnaire contained several brief attention-check questions. We also used three validated tools, and before launching the survey, the questionnaire was piloted with ten caregivers. Moreover, to ensure that respondents will provide valid and accurate data, we have established clear and strict criteria for participation, and the DBMD patient organization, StopDuchenne Foundation, supported the recruitment. Additionally, all respondents were informed about your data collection’s purpose, scope, methods and duration. Finally, the data was monitored and controlled for completion rate and data quality throughout the entire data collection process.

The survey was conducted for six months, between March and August 2024. After the letter with the invitation to participate in the study was sent to several advocacy organizations and support groups for DBMD patients and their families and permission was obtained, the principal investigator (JD) posted the link to an online questionnaire on their Facebook pages and all DBMD caregivers were invited to participate. However, before completing the survey, all eligible participants were requested to fill out a written informed consent form posted at the beginning of the study. Every caregiver who volunteered to participate provided written online informed consent before completing the survey. Financial compensation was not provided for taking part in the study. The survey took about 15–20 min to complete.

### Participants

The eligibility requirements for the study were being over 18 years old, being a parent or family caregiver of an individual with DMD or BMD, giving direct care to a person with a confirmed molecular diagnosis of DMD or BMD, speaking Polish, having access to the Internet and having the capability to use electronic devices to engage in an online survey, and providing written informed consent to participate.

### Questionnaires

The survey questionnaire used in this study comprised four parts. The first section contained single-choice, closed-ended questions about the sociodemographic characteristics of caregivers and DBMD patients. The second section included the abbreviated Polish version of the WHO Quality of Life-BREF (WHOQOL-BREF), known for its reliability [57]. While it is often used in clinical trials and epidemiological studies, this tool is broken into 24 items divided into four distinct domains: physical health (7 questions on mobility, daily activities, functional capacity, energy, pain, and sleep; score range: 7–35), psychological (6 questions on one’s self-image, negative thoughts, positive attitudes, self-esteem, mentality, learning ability, memory concentration, religion, and the mental status; score range: 6–30), social relationships (3 questions on personal relationships, social support, and sex life; score range: 3–15), and environmental (8 questions on financial resources, safety, health and social services, living physical environment, opportunities to acquire new skills and knowledge, recreation, general environment, and transportation; score range: 8–40). It also comprises two questions assessing respondents’ overall quality of life and health satisfaction. While answering these questions, respondents used a 5-point Likert scale according to their experiences over the previous two weeks. Higher ratings indicate a higher quality of life.

The third section contained the Polish version of the Zarit Burden Interview (ZBI) [58]. This self-administered tool assesses how providing care affects a person’s psychological well-being, financial situation, relationships between the caregiver and the care recipient, and social life. Each of the 22 items is given a 5-point Likert-type rating, and the total score, which can range from 0 to 88, is the result of adding all of the scores. Higher ratings indicate an increased level of caregiver stress.

The final section of the questionnaire consisted of the Consumer Financial Protection Bureau Financial Well-Being Scale (CFPB-FWBS) [59]. This freely available tool contains ten questions to assess a person’s freedom of choice and financial security. Since the CFPB-FWBS did not exist in Polish, it was back-translated by two translators proficient in English and Polish and then adapted to Polish circumstances. Two groups of twenty people each were used for the tool’s pre-testing: a group of caregivers for children with chronic illnesses and a separate group of healthy adults. Even in such small groups, the scale proved highly sensitive and consistent, confirming its suitability for our investigation.

### Data analysis

The collected data was meticulously reviewed for accuracy, consistency, and completeness before being processed and analyzed using JASP 0.18.3 and PQStat 1.8.4 for comprehensive statistical analysis. The results are presented through descriptive statistics. Logistic regression models were applied to evaluate respondents’ quality of life (QoL) differences across the four WHOQOL domains and the ZBI. Sociodemographic factors were used as explanatory variables, with the age of the DBMD patient and the CFPB-FWBS score as covariates. National average values [57] served as cut-off points within the WHOQOL domains, dividing caregivers into two groups: those scoring below and those scoring above the average. Kruskal-Wallis ANOVA was used to analyze ZBI and WHOQOL domain results based on CFPB financial well-being groups, with post-hoc Conover-Iman tests employed for group comparisons. A p-value of less than 0.05 was considered statistically significant.

## Results

### Caregivers’ and DBMD patients’ characteristics

Table 1 presents the socio-demographic characteristics of 202 caregivers of children with DBMD. Most caregivers were women (85.6%) and between the ages of 30–49 (90.6%). While 85.1% of caregivers were mothers, 77.7% provided care with a partner or spouse. Over half (53.9%) held a university degree, and 36.1% lived in areas with under 10,000 inhabitants. Over half (55%) were unemployed due to childcare responsibilities, while 34.7% were employed full-time.

**Table 1:** Socio-demographic characteristics of DBMD caregivers’

Characteristics	*N* (%)
Total respondents	202 (100)
**Sex**	
Women	173 (85.6)
Men	29 (14.4)
**Age (in years)**	
20–29	3 (1.5)
30–39	95 (47)
40–49	88 (43.6)
50–59	14 (6.9)
60–69	2 (1)
**Relationship with a person with DBMD**	
Mother	172 (85.1)
Father	28 (13.9)
Grandmother	1 (0.5)
Another family member	1 (0.5)
**I provide care for the patient**	
Alone	22 (10.9)
With a partner/spouse	157 (77.7)
With the help of family, extended relatives, social institution	23 (11.4)
**Education**	
Primary school	3 (1.5)
Vocational school	29 (14.4)
High school	61 (30.2)
University	96 (47.5)
Medical university	13 (6.4)
**Domicile**	
Up to 10,000 inhabitants	73 (36.1)
10–50,000 inhabitants	44 (21.8)
51–100,000 inhabitants	26 (12.9)
101–500,000 inhabitants	24 (11.9)
Above 500,000 inhabitants	35 (17.3)
**Professional activity**	
Unemployed	3 (1.5)
Unemployed due to caregiving	111 (55)
Employed part-time	16 (7.9)
Employed full-time	70 (34.7)
Pension	2 (1)

Table 2 summarizes the demographic and diagnostic characteristics of patients with DBMD. Of the 202 patients, 99% were male, with an age range of 1 to 31 years (mean age: 10.4 years, SD: 5.6 years, median: 10 years). The majority were diagnosed between the ages of 2–5 years (61.4%), while 1.5% had prenatal diagnoses. Diagnostic tests were primarily funded by the National Health Fund (81.2%), with 12.4% of families using their financial resources and a smaller portion (3.5%) through participation in research programs.

**Table 2:** DBMD patient’s characteristics

Characteristics	*N* (%)
**Sex**	
Female	2 (1)
Male	200 (99)
**Age** (in years)	
Range	1–31
Mean	10.4
SD	5.6
Median	10
**Age of diagnosis**	
0–1 year	40 (19.8)
2–3	57 (28.2)
4–5	67 (33.2)
6–10	36 (17.8)
11–15	2 (1)
**Source of funding for diagnostic tests**	
The National Health Fund	164 (81.2)
Participation in experimental research, university/hospital research grant	7 (3.5)
Own financial resources	25 (12.4)
Private and external help	6 (3)

### DBMD caregivers’ quality of life, caregiving burden and financial well-being

Table 3 presents descriptive statistics for the WHOQOL domains, ZBI, and CFPB-FWBS. For the WHOQOL physical health domain, scores ranged from 2.14 to 17.14, with a mean of 10.43 (SD = 3.03) and 85.1% of respondents scoring below the national average. In the WHOQOL psychological domain, scores ranged from 0 to 19.7, with a mean of 10.58 (SD = 3.52) and 72.3% of respondents below the national average. The WHOQOL social relationships domain showed a mean of 10.48 (SD = 3.91), with 82.2% below the national average. The WHOQOL environmental domain’s mean was 9.92 (SD = 2.97), and 83.7% scored below the national average.

The ZBI had a mean score of 35.3 (SD = 12.5), and 67.8% scored below the burnout threshold. The CFPB-FWBS had a mean of 48.8 (SD = 11.8), with the majority of respondents in the medium-low (35.1%) and medium-high (27.7%) financial well-being groups.

**Table 3:** Descriptive statistics for WHOQOL domains, ZBI, and CFPB-FWBS

Scale	Descriptive statistic
**WHOQOL physical health domain**	
Range	2.14–17.14
Mean (SD)	10.43(3.03)
Median	10
Mean country score [95%CI]	14.39 [14.21–14.57]
Number of respondents with a score below the mean country score N (%)	172 (85.1)
**WHOQOL psychological domain**	
Range	0-19.7
Mean (SD)	10.58 (3.52)
Median	10.83
Mean country score [95%CI]	13.13 [12.95–13.30]
Number of respondents with a score below the mean country score N (%)	146 (72.3)
**WHOQOL social relationships domain**	
Range	0–20
Mean (SD)	10.48 (3.91)
Median	10
Mean country score [95%CI]	14.09 [13.89–14.29]
Number of respondents with a score below the mean country score N (%)	166 (82.2)
**WHOQOL environmental domain**	
Range	1.25–18.75
Mean (SD)	9.92 (2.97)
Median	10
Mean country score [95%CI]	12.91 [12.76–13.07]
Number of respondents with a score below the mean country score N (%)	169 (83.7)
**ZBI**	
Range	4–74
Mean (SD)	35.3(12.5)
Median	35
Number of respondents with a score below the value recognized as the burnout threshold	137 (67.8)
**CFPB-FWBS**	
Range	14–86
Mean (SD)	48.8 (11.8)
Median	49.5
**Score ranges divided by groups based on financial well-being N (** **%)**	
Very low	13 (6.4)
Low	17 (8.4)
Medium-low	71 (35.1)
Medium-high	56 (27.7)
High	34 (16.8)
Very high	11 (5.4)

### Factors associated with caregivers’ quality of life, caregiving burden and financial well-being

Table 4 presents the results of the regression analysis for the WHOQOL domains. Financial well-being (CFPB-FWBS) was a significant predictor across all models, with odds ratios (OR) ranging from 1.072 to 1.124, indicating a strong association with higher WHOQOL domain scores. The patient’s age significantly affected the WHOQOL psychological domain (Model 2.2 - OR = 1.093, *p* < 0.01; Model 2.3 - OR = 1.079, *p* < 0.05). University education was associated with worse outcomes in the WHOQOL social relationships domain (OR = 0.444, *p* < 0.05). The models explained 9–28% of the variance, with McFadden’s R² ranging from 0.091 to 0.204 and Nagelkerke R² ranging from 0.135 to 0.282. All models for the four WHOQOL domains were highly significant (*p* < 0.001), while others showed varying significance levels. The results emphasize the strong impact of financial well-being on caregivers’ quality of life, especially in the psychological and environmental domains.

**Table 4:** Regression analysis results for WHOQOL domains

Regression parameters	WHOQOL physical health	WHOQOL psychological				WHOQOL social relationships		WHOQOL environmental
	Model 1.1	Model 1.2	Model 2.1	Model 2.2	Model 2.3	Model 3.1	Model 3.2	Model 4.1
	OR (SE)	OR (SE)	OR (SE)	OR (SE)	OR (SE)	OR (SE)	OR (SE)	OR (SE)
Intercept	0.002^***^ (1.139)	9.125 × 10^-4***^ (1.303)	0.006^***^ (0.930)	0.001^***^ (1.109)	0.001^***^ (1.142)	0.006^***^ (0.990)	0.005^***^ (1.006)	3.949 × 10^-4***^ (1.325)
CFPB-FWBS	1.089^***^ (0.020)	1.093^***^ (0.021)	1.087^***^ (0.017)	1.096^***^ (0.018)	1.111^***^ (0.020)	1.072^***^ (0.018)	1.077^***^ (0.019)	1.124^***^ (0.023)
Age of a patient		1.056 (0.037)		1.093^**^ (0.031)	1.079^*^ (0.032)			
University education vs. other					0.444^*^ (0.394)			
Male vs. Female							0.335 (0.688)	
Providing care independently vs. others								
Non-working vs. working								
Observations	202	202	202	202	202	202	202	202
R2 McFadden	0.127	0.139	0.124	0.160	0.178	0.091	0.107	0.204
R2 Nagelkerke	0.178	0.194	0.196	0.248	0.274	0.135	0.157	0.282
AIC	152.202	152.109	212.933	206.339	203.956	176.088	175.010	147.155
p-value for Model	< 0.001	0.148	< 0.001	0.003	0.036	< 0.001	0.079	< 0.001

Notes * p-value < 0.05; ** p-value < 0.01; *** p-value < 0.001

Table 5 presents the results of two logistic regression models predicting caregivers’ burden (ZBI score). Model 5.1 includes the CFPB variable, while Model 5.2 incorporates an additional parameter, the caregiver’s education level. Both models show statistically significant relationships between CFPB and ZBI scores, with CFPB negatively associated with caregiver burden (*p* < 0.001). Model 5.2 highlights that university-educated caregivers report a higher burden than those with lower education levels (OR = 2.529, *p* < 0.01*). The fit statistics, including McFadden’s R² and Nagelkerke R², indicate moderate explanatory power, with Model 5.2 showing an improvement over Model 5.1. AIC values suggest that Model 5.2 provides a better fit to the data. Both models are statistically significant (*p* < 0.001 and *p* = 0.006).

**Table 5:** Regression analysis results for ZBI

Regression parameters	ZBI	
	Model 5.1	Model 5.2
	OR (SE)	OR (SE)
Intercept	5.301^*^ (0.695)	5.961^*^ (1.006)
CFPB-FWBS	0.951^***^ (0.014)	0.938^***^ (0.016)
Age of a patient		
University education vs. other		2.529^**^ (0.344)
Male vs. Female		
Providing care independently vs. others		
Non-working vs. Working		
Observations	202	202
R2 McFadden	0.054	0.084
R2 Nagelkerke	0.092	0.140
AIC	244.094	238.434
p-value for model	< 0.001	0.006

Notes * p-value < 0.05; ** p-value < 0.01; *** p-value < 0.001

Table 6 presents the Kruskal-Wallis ANOVA analysis results comparing the ZBI and WHOQOL domain scores across six groups of caregivers categorized by financial well-being. Significant differences were observed between the groups for all measures. The ZBI scores ranged from 4 to 74, with a mean score of 40 for those with very low financial well-being and 26.5 for those with very high financial well-being (H = 16.876, *p* < 0.01). For the WHOQOL domains, higher financial well-being consistently correlated with better outcomes. In the WHOQOL physical health domain, mean scores ranged from 8.35 for very low well-being to 13.64 for very high well-being (H = 38.326, *p* < 0.001). Similar trends were noted in the psychological, social relationships, and environmental domains, with p-values indicating highly significant group differences in all comparisons, with the highest difference observed in the environmental domain. The results highlight a clear association between financial well-being and both caregiver burden and quality of life, emphasizing the impact of financial stability on these measures.

**Table 6:** Kruskal-Wallis ANOVA analysis of the results of Zarit Burden Interview and WHOQOL domains divided according to CFPB Financial Well-being scale groups

	Financial well-being					
	1. Very low	2. Low	3. Medium-low	4. Medium-high	5. High	6. Very high
Valid	13	17	71	56	34	11
**Zarit Burden Interview**						
Range	23–56	15–71	14–68	13–56	10–74	4–48
Median	39	42	36	35	29.5	26
Mean	40	40.9	37.3	34.3	31.1	26.5
SD	10.8	16	11.7	9.8	14.5	12.1
Kruskal-Wallis ANOVA	H = 16.876**					
p for group differences	1 vs.5* 1 vs.6**	2 vs.5** 2 vs.6**	3 vs.5** 3 vs.6*		5 vs.1* 5 vs.2** 5 vs.3**	6 vs.1** 6 vs.2** 6 vs.3*
**WHOQOL physical health**						
Range	5.71–12.14	4.29–14.29	4.29–15.71	2.14–15.71	3.57–17.14	7.86–17.14
Median	8.57	7.14	10	10.71	11.79	14.29
Mean	8.35	7.98	10.13	10.56	11.83	13.64
SD	1.76	2.54	2.31	2.93	3.62	2.92
Kruskal-Wallis ANOVA	H = 38.326***					
p for group differences	1 vs.3* 1 vs.4** 1 vs.5*** 1 vs.6***	2 vs.3** 2 vs.4*** 2 vs.5*** 2 vs.6***	3 vs.1* 3 vs.2** 3 vs.5** 3 vs.6***	4 vs.1** 4 vs.2*** 4 vs.6**	5 vs.1*** 5 vs.2*** 5 vs.3**	6 vs.1*** 6 vs.2*** 6 vs.3*** 6 vs.4**
**WHOQOL psychological**						
Range	4.17–12.50	3.33–13.33	3.33–16.67	0.833–17.50	0-17.50	2.50–9.17
Median	7.50	10	10	11.25	13.33	14.17
Mean	7.89	9.31	10.24	10.82	11.59	13.56
SD	2.37	2.94	2.80	3.46	4.19	4.86
Kruskal-Wallis ANOVA	H = 25.259***					
p for group differences	1 vs.3** 1 vs.4** 1 vs.5*** 1 vs.6***	2 vs.5** 2 vs.6**	3 vs.1** 3 vs.5** 3 vs.6**	4 vs.1** 4 vs.6*	5 vs.1*** 5 vs.2** 5 vs.3**	6 vs.1*** 6 vs.2** 6 vs.3** 6 vs.4*
**WHOQOL social relationships**						
Range	5–15	0-16.67	0-16.67	3.33-20	0–20	6.67-20
Median	8.33	10	10	11.67	12.50	15
Mean	8.85	9.41	9.62	10.86	11.72	13.9
SD	2.75	4.29	3.22	4.06	4.11	4.54
Kruskal-Wallis ANOVA	H = 18.782**					
p for group differences	1 vs.4* 1 vs.5** 1 vs.6**	2 vs.6*	3 vs.5** 3 vs.6**	4 vs.1* 4 vs.6*	5 vs.1** 5 vs.3**	6 vs.1** 6 vs.2* 6 vs.3** 6 vs.4*
**WHOQOL environmental**						
Range	1.25-10	3.75–10.63	5-15.63	3.13–15.63	6.88–16.25	6.25–18.75
Median	7.50	8.13	9.38	10.63	12.50	13.75
Mean	6.30	7.35	9.32	10.25	12.15	13.52
SD	2.66	1.97	2.15	2.53	2.58	3.30
Kruskal-Wallis ANOVA	H = 65.863***					
p for group differences	1 vs.3*** 1 vs.4*** 1 vs.5*** 1 vs.6***	2 vs.3** 2 vs.4*** 2 vs.5*** 2 vs.6***	3 vs.1*** 3 vs.2** 3 vs.4** 3 vs.5*** 3 vs.6***	4 vs.1*** 4 vs.2*** 4 vs.3** 4 vs.5** 4 vs.6**	5 vs.1*** 5 vs.2*** 5 vs.3*** 5 vs.4**	6 vs.1*** 6 vs.2*** 6 vs.3*** 6 vs.4**

Notes * p-value < 0.05; ** p-value < 0.01; *** p-value < 0.001

## Discussion

While previous research demonstrated that providing care for an individual with DBMD affects the physical and mental well-being of caregivers, their emotional state, family relationships and social interactions, and financial load [18, 20, 38–40], four significant conclusions come from this study, which add to the body of knowledge regarding the relationship between parental stress and caregiving a person with DBMD.

First, it shows that while DBMD significantly affects caregivers’ quality of life, they score significantly lower than the national average. Moreover, compared to the national mean score, DBMD caregivers enrolled in this study performed far worse in every measured domain: in the physical health domain, 85.1% of caregivers scored below the national average; in the environmental domain, it was 83.7%; in the social relationships domain, 82.2%; and in the psychological domain, 72.3%. At the same time, this study confirms that as DBMD affects every aspect of caregivers’ lives, it is their physical health and financial well-being that are affected the most, followed by the deterioration of social life and psychological state. However, a recent study by Ishizaki et al. also reported that Japanese caregivers’ health-related quality of life was somewhat lower than in the general population, especially in physical functioning, role limitations-physical, bodily pain, and social [17]. Studies also reported that many caregivers experience deterioration in physical health, pain, and disturbed sleep [18, 41–44]. A significant psychological and emotional strain is also common [18, 41–44]. Significantly, while Landfeldt et al. observed that half of DBMD caregivers reported moderate to severe anxiety or depression, these rates were far higher than those of the general population. Moreover, while 12–40% of caregivers believed they should be doing more for their DBMD children, many others worried about the future of their child (57–86%), had problems combining caregiving and other family or work responsibilities (26–69%), and were concerned about not having enough money to take care of their son (17–62%) [42].

Secondly, this research shows that DBMD is a source of severe burden for caregivers. This is in line with previous findings showing that although providing care for persons with DBMD can be meaningful, rewarding and fulfilling, it is also a source of a significant subjective caregiver load [60]. It also confirms that caregivers’ quality of life was strongly related to their perceived burden [38]. For example, while 56% of Brazilian caregivers reported a feeling of burden [38], in a study by Kenneson and Bobo, 46.4% of women reported a high level of stress related to caring for a relative with DBMD, only 61.7% were satisfied with their lives, and 50.4% experienced a high level of burden [18]. At the same time, in our study, the average for ZBI was 35.3, implying a mild-to-moderate burden; it was somewhat higher than in other counties. For example, while French caregivers reported a mean physical and emotional burden of 23.4 [15], among Japanese caregivers, it was 20.9 [17], and in Brazil, 26.3 [38]. Similarly, a multinational study in Germany, Italy, the United Kingdom, and the United States reported caregivers’ burden score at 29 [42]. Finally, while another international study reported that the mean burden score was 28.3, some differences between various countries were also observed, as in France it was 14.5; in Italy, 24.5; in Spain, 32.5; in the United Kingdom 31.3; in Germany 34.7, and in Sweden 37.3 [61].

While DBMD is usually diagnosed by age 5, it was demonstrated that the gap between the first onset of symptoms and the final diagnosis is approximately two and a half years [62, 63]. Consequently, since prompt genetic counselling and corticosteroid therapy initiation are lost due to this diagnostic delay, it is an important factor that increases caregivers’ subjective burden [64]. For example, it was demonstrated that in their quest for a diagnosis, parents sought advice from a variety of medical specialists and went through a spectrum of emotions. Moreover, while their child’s disease severely stresses them, many parents feel frustrated because they believe that their child could have received a diagnosis sooner [65, 66]. At the same time, caregivers of persons with DBMD experience high levels of financial strain related to both indirect costs associated with lost work productivity, absenteeism, travel time to visit hospitals, and out-of-pocket caregiving-related expenditures [67] and direct costs of illness associated with healthcare utilization, i.e. medical treatments, therapy, the need to purchase assistive devices, including wheelchair, and home modifications. Managing these costs can severely impact caregivers’ financial stability and limit their ability to prioritize self-care or access resources for their well-being [61, 68, 69]. Significantly, while compared to other diseases, both caregivers of younger and adult persons with DBMD experience higher levels of burden and financial strain, it is the latter who are more burdened. Research showed that while transitioning from childhood to adulthood is emotionally challenging for caregivers [22–24], due to prolonged care, caregivers are burdened by the lack of legal regulation, social services and education system for their adult persons with DBMD. Moreover, the shift in health care from pediatric to adult is particularly challenging for caregivers [22, 70].

Thirdly, although caregivers reported varying levels of financial well-being, it is important to note that most of the respondents had to give up work to care for their DBMD relatives. Financial well-being emerged as the most significant predictor of respondents’ quality of life (particularly in the psychological and environmental health domains) and caregiving burden [16, 61, 71]. For example, while Kenneson and Bobo reported that 50% of DBMD caregivers revealed a high burden and nearly 40% were dissatisfied with their lifestyles, they also reported that limited income, social support, and low resilience were associated with distress and high levels of stress. High-stress levels were also predicted by having a job outside the home [18].

In Germany, the estimated yearly disease burden, including direct medical/non-medical, indirect and informal care costs for BMD, were estimated at €39,060, and DMD was almost twice as high at €78,913 [21]. In Portugal, according to 2019 figures, the average patient stage-specific DMD expenses were estimated at €19,993 in the ambulant stage and €48,991 in the non-ambulant stage [72]. At the same time, while significant differences between various countries were observed, Hungary had the lowest average annual cost per DMD person (€7657), while France had the highest (€58,704) [61]. A similar burden was in the US, where healthcare costs for DMD increased tenfold as compared to the group of other patients with RDs ($23,005 vs. $2 277), primarily due to higher medical costs (difference of $22,000) [52]. Finally, in Australia, the average cost of healthcare for a person with DMD was determined at $10,046, which was much more than the average cost of healthcare in Australia for all age groups. Healthcare costs accounted for 22% of the overall costs, with a mean of $46,700 (median $32,300) [51].

On the other hand, Soelaeman et al. reported that compared to caregivers of children without the disease, parents of boys with DBMD worked an average of 296 fewer hours annually, which translates to an $8816 reduction in earnings in 2020 U.S. dollars [50]. Similarly, according to Schreiber-Katz et al., to care for their child, 29% of parents of boys with DMD in Germany quit their jobs, and 38% of working parents had to reduce their working hours by an average of 15 h per week. Meanwhile, only 4% of parents of boys with BMD quit their jobs and only 12% reduced working hours [21].

Fourthly, this study shows that apart from financial well-being, some dimensions of respondents’ quality of life and perceived burden were also associated with patients’ age and caregivers’ educational status. Thus, although some studies found no evidence that the age of children with DMD affected the stress and anxiety levels of their caregivers [44], our results are in line with previous studies showing that while the transition period from childhood to adulthood is a source of many emotional, financial and healthcare burdens [22–24], caregivers’ burden is negatively correlated with patients’ age and number of years spent on caregiving [38]. Additionally, although some research suggests that one of the predictors of caregivers’ stress was employment outside of the home (by being apart from a special needs child) [18], Landfeldt et al. identified a significant correlation between the caregivers’ anxiety and depression and their perception of patients’ physical and mental health and objective burden, such as the number of hours of leisure time spent providing informal care and the annual household cost burden [42]. Moreover, the international study demonstrated that between 27 and 49% of caregivers reduced their working hours or had to quit their jobs. Moreover, the mean number of hours of free time spent providing informal care each week was between 33 and 44 [68].

### Limitations

Even though this is one of the first Polish studies examining the caregiving experiences of DBMD parents, it has some limitations. While a reasonably high number of 202 carers completed the survey, since the number of DBMD patients in the country is estimated at 800–1000, this number could be higher. However, it should be stressed that the exact number of persons with DBMD in Poland is unknown due to a lack of a patient registry. There is a risk of recruitment bias since the data was collected with the help of an online advocacy group for patients and their families, the StopDuchanne Foundation and the Salamander Foundation, via their Facebook pages. Thus, parents who are not affiliated with the Foundations or do not use the Internet might have limited access to the survey. Additionally, although 14.4% of respondents were males, female caregivers still predominated. Since we did not collect information about the natural history of patients’ diseases, this methodology’s self-reported, subjective, and retrospective nature limits the outcomes. Similarly, due to their career status, some DBMD caregivers may experience their own cardiac or muscle problems and have a disproportionately high care burden. Since this study focussed on the challenges related to caregiving, we did not directly gauge the benefits and positive aspects of providing care. Lastly, more comprehensive qualitative research is needed to better understand caregivers’ experiences with daily challenges and needs in DBMD.

## Conclusions

Given that this study has pinpointed that providing care for a person with DBMD significantly affects caregivers’ every aspect of quality of life, including physical health, financial, social and psychological well-being, and is a source of significant subjective burden for caregivers, their employment requires involving the application of multilayer strategies. At the same time, it seems that to improve DBMD caregivers’ quality of life and alleviate their feeling of caregiver burden, future intervention programs should focus on promoting resiliency and active coping and developing a social support system and respite care.

At the same time, since most DBMD caregivers reported a significant financial strain and economic well-being was significantly correlated with their quality of life and caregiver burden to fulfil their needs, it is crucial to provide caregivers with adequate financial support and resources.

## Data Availability

The data supporting this study’s findings are not openly available due to reasons of sensitivity and are available from the corresponding author upon reasonable request. Data are located in controlled access data storage at Poznan University of Medical Sciences.

## Abbreviations

CFPB-FWBS: The Consumer Financial Protection Bureau’s Financial Well-Being Scale
DMD: Duchenne muscular dystrophy
WHOQOL-BREF: World Health Organization Quality of Life-BREF
